# A new protocol for exercise testing in COPD; improved prediction algorithm for *W_MAX_* and validation of the endurance test in a placebo-controlled double bronchodilator study

**DOI:** 10.1177/17534666211037454

**Published:** 2021-09-30

**Authors:** Ellen Tufvesson, Finn Radner, Anton Simonsen, Georgia Papapostolou, Linnea Jarenbäck, Saga Jönsson, Ulf Nihlen, Alf Tunsäter, Jaro Ankerst, Stefan Peterson, Leif Bjermer, Göran Eriksson

**Affiliations:** Respiratory Medicine and Allergology, Department of Clinical Sciences, Lund, Lund University, Skane University Hospital, 221 85 Lund, Sweden; Respiratory Medicine and Allergology, Department of Clinical Sciences, Lund, Lund University, Skane University Hospital, Lund, Sweden; Respiratory Medicine and Allergology, Department of Clinical Sciences, Lund, Lund University, Skane University Hospital, Lund, Sweden; Respiratory Medicine and Allergology, Department of Clinical Sciences, Lund, Lund University, Skane University Hospital, Lund, Sweden; Respiratory Medicine and Allergology, Department of Clinical Sciences, Lund, Lund University, Skane University Hospital, Lund, Sweden; Respiratory Medicine and Allergology, Department of Clinical Sciences, Lund, Lund University, Skane University Hospital, Lund, Sweden; Respiratory Medicine and Allergology, Department of Clinical Sciences, Lund, Lund University, Skane University Hospital, Lund, Sweden; Respiratory Medicine and Allergology, Department of Clinical Sciences, Lund, Lund University, Skane University Hospital, Lund, Sweden; Respiratory Medicine and Allergology, Department of Clinical Sciences, Lund, Lund University, Skane University Hospital, Lund, Sweden; Regional Cancer Centre South, Region Skane, Lund, Sweden; Respiratory Medicine and Allergology, Department of Clinical Sciences, Lund, Lund University, Skane University Hospital, Lund, Sweden; Respiratory Medicine and Allergology, Department of Clinical Sciences, Lund, Lund University, Skane University Hospital, Lund, Sweden

**Keywords:** COPD, endurance time, exercise testing, lung function, prediction, *W_MAX_*

## Abstract

**Background:**

Two new protocols have been developed for bicycle exercise testing in chronic obstructive pulmonary disease (COPD) with an individualized cardiopulmonary exercise test (ICPET) and subsequent customized endurance test (CET), which generate less interindividual spread in endurance time compared with the standard endurance test. Main objectives of this study were to improve the prediction algorithm for *W_MAX_* for the ICPET and validate the CET by examining treatment effects on exercise performance of indacaterol/glycopyrronium (IND/GLY) compared with placebo.

**Methods:**

COPD patients, with forced expiratory volume in 1 s (*FEV_1_*) 40–80% predicted, were recruited. Pooled baseline data from two previous studies (*n* = 38) were used for the development of an improved *W_MAX_* prediction algorithm. Additional COPD patients (*n* = 14) were recruited and performed the ICPET, using the new prediction formula at visit 1. Prior to the CET at visits 2 and 3, they were randomized to a single dose of IND/GLY (110/50 µg) or placebo.

**Results:**

The improved multiple regression algorithm for *W_MAX_* includes diffusing capacity for carbon monoxide (*DLCO*), *FEV_1_*, *sex*, *age* and *height* and correlated to measured *W_MAX_* (*R*^2^ = 0.89 and slope = 0.89). Treatment with IND/GLY showed improvement in endurance time *versus* placebo, mean 113 s [95% confidence interval (CI): 6–220], *p* = 0.037, with more prominent effect in patients with *FEV*_1_ < 70% predicted.

**Conclusion:**

The two new protocols for ICPET (including the new improved algorithm) and CET were retested with consistent results. In addition, the CET showed a significant and clinically relevant prolongation of endurance time for IND/GLY *versus* placebo in a small number of patients.

## Introduction

Impaired exercise tolerance is a major problem in patients with chronic obstructive pulmonary disease (COPD) with usually multifactorial causes including airflow limitation, reduced gas exchange, peripheral muscle weakness and coronary heart disease.^1–3^ It is associated with poor prognosis and impaired quality of life.^4^ A bicycle constant work rate exercise test (CWRET) at 75–80% of peak maximum workload (*W_MAX_*) is frequently used to assess the impact of COPD on functional capacity and the response to medical treatment or pulmonary rehabilitation.^5–11^ Endurance time is a valuable outcome because it is related to multiple clinical aspects of disease severity in COPD,^12^ and measured at a ‘standard’ CWRET, it would be most sensitive to interventions when it lasts between 3 and 8 min.^7,13,14^ According to the American Thoracic Society/European Respiratory Society Task force, an improvement of 46–105 s can be considered clinically important.^15^ A limitation of the standard CWRET is the variation in endurance time among subjects. Reducing the interindividual variability in endurance time would allow smaller sample sizes and less costly clinical trials whose results may also be easier to interpret.^7^

A standard CWRET is preceded by an incremental bicycle test designed to measure the patient’s maximum peak work rate (*W_MAX_*). A standard maximum test, which frequently uses a protocol similar to a cardiopulmonary exercise test (CPET),^16^ may lead to either an underestimation or overestimation of *W_MAX_*, leading to a too long or short endurance time with the standard CWRET.

We embarked on a development programme with the objective to improve the protocol for the *W_MAX_* test and the standard CWRET. In the first of our studies^17^ in patients with moderate to severe COPD, a protocol for a new, individualized, *W_MAX_* test was developed. This test will hereafter be denoted individualized cardiopulmonary exercise test (ICPET). During the ICPET, patients started cycling for 3 min at a workload of 40% of a predicted *W_MAX_*, calculated for each patient using a random forest model based on multicentre industry data. This part of the test was followed by a linear increase in load, estimated to reach predicted *W_MAX_* after an additional 8 min. Predicted values of *W_MAX_* correlated well with measured *W_MAX_*. However, the value for the slope for predicted *versus* measured *W_MAX_* was 0.50, which indicated that *W_MAX_* values in the high range (high performers) and the low range (low performers) were underestimated and overestimated, respectively. In Tufvesson and colleagues,^18^ two different new test protocols for measuring bicycle exercise endurance time were compared with results from a standard CWRET. Both studies started with an initial period at 30–40% of *W_MAX_*. In the first new protocol, the endurance period started at 75% of *W_MAX_*, and thereafter, the workload increased stepwise until exhaustion. The second new protocol started at 70% of *W_MAX_* and thereafter increased in a linear fashion until exhaustion. Both protocols resulted in reductions of the standard deviation and the range of endurance time compared with the standard CWRET. Overall, the second new protocol showed more advantageous properties than the first when compared with results from the standard CWRET.^18^ The second protocol served as a model for the design of the protocol used for the endurance test in this study, hereafter called the customized endurance test (CET).

The main objectives of this study were twofold: Part 1: to improve the prediction algorithm for *W_MAX_* to be used for the ICPET by using combined data from study cohorts included in our previous studies.^17,18^ Part 2: to study the protocols for both the ICPET and the subsequent CET, the latter by examining the effect of the double bronchodilator [long-acting β_2_-agonist (LABA) plus long-acting muscarinic antagonist (LAMA), i.e. indacaterol and glycopyrronium (IND/GLY)] *versus* placebo on exercise performance.

## Materials and methods

### Studies

The development programme to find a new exercise test consisted of three elements in three studies; A, B and C (this study):

- Prediction algorithm: A prediction algorithm was constructed from baseline data derived from a multicentre study^6^ and was used in studies A and B.^17,18^ A new prediction algorithm was constructed based on baseline data from studies A and B, and applied in study C (see below).
- ICPET: In study A, an ICPET with a linear increase of workload until exhaustion (*W_MAX_*) was used. The ICPET in study A was compared with a standard, stepwise CPET.^17,18^ In studies B^18^ and C (see below), the same ICPET with minor changes was used.
- CET: In study A, the CET used a stepwise increase of workload until exhaustion. The increase was deemed too aggressive and in study B, the CET was changed to a linear and lower increase of workload until exhaustion. Both studies A and B included a standard CWRET as control.^18^ In this study, the same linear CET as in study B (with some minor changes, see below) was used.

### Patients

All participants in the studies A to C had a COPD diagnosis and a postbronchodilator *FEV*_1_ of 40–80% of predicted normal (%pred) and a ratio of *FEV*_1_ to forced vital capacity (*FVC*) of ⩽0.7. No other lung function criteria were applied for selection of the study population. Patients with a history of COPD exacerbation within 6 months prior to the study or with cardiovascular disease or any other condition that was considered to put the patients at increased risk or interfere with the examinations in the study were excluded.

### Ethics

The Regional Ethical Review Board in Lund, Sweden, approved the studies, which complied with the Declaration of Helsinki. Approval number: 2013/850. Written informed consent was obtained from all participants prior to any study-related procedure.

### Part 1

#### Improvements of the prediction algorithm (studies A and B)

Pooled baseline data from studies A and B^17,18^ were used for the development of an improved prediction algorithm for *W_MAX_* using multiple regression as described in the statistical section (for a flowchart of the patients, see Figure S1 in the Online Supplement).

### Part 2

#### Bronchodilator effect on exercise performance (study C)

Study C was a single-centre, double-blind, randomized, three-visit crossover trial. Visit 1 started with a screening examination including collection of demographic data together with medical and smoking history, COPD medications and concomitant medications. Furthermore, the participants answered the Clinical COPD Questionnaire (CCQ)^19^ and underwent a physical examination including vital signs and a 12-lead electrocardiogram (ECG). A physician assessed the patient’s clinical status and approved or disapproved further participation. Starting 15 min after inhalation of 400-µg salbutamol, lung function was measured with spirometry, body plethysmography and diffusing capacity for carbon monoxide (*DLCO*). Thereafter, the participants performed an ICPET. The predicted *W_MAX_* used at the ICPET was calculated by the new improved prediction algorithm.

**Figure 1.**
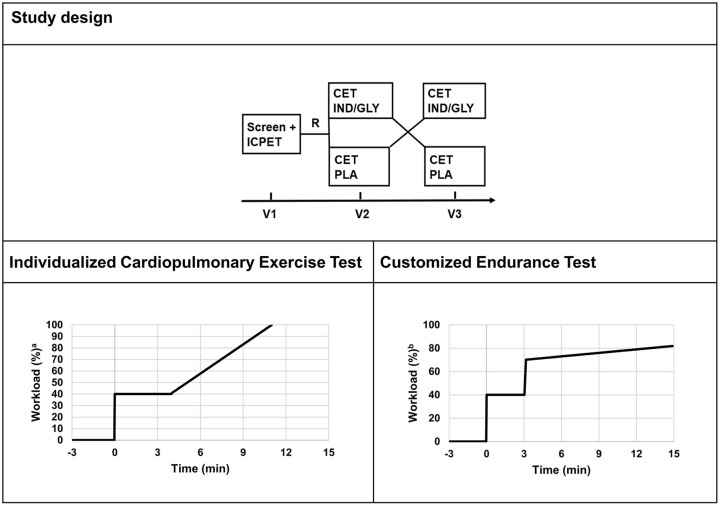
Schematic study design of study C and schematic designs of the ICPET and the CET used. CET, customized endurance test; ICPET, individualized cardiopulmonary exercise test; IND/GLY, indacaterol/glycopyrronium; PLA, placebo. ^a^Workload in % of predicted *W_MAX_* value. ^b^Workload in % of maximum value from the ICPET.

Both visits 2 and 3 started with a physical examination including vital signs, ECG, spirometry and answering of the CCQ. Participants who were considered, by the study physician, to be eligible to perform a CET were randomized (at visit 2) to inhale the study medication [single dose of IND/GLY 110/50 µg (Novartis Pharma AG, Basel, Switzerland) or placebo] 60 min prior to the CET, and vice versa at visit 3. For an overview of the study design, see Figure 1.

The participants were to change their LABA and/or LAMA regimens to a short-acting muscarinic antagonist (SAMA) 3–4 times a day at least 48 h prior to visits 2 and 3. In addition, all participants received a short-acting β_2_-agonist (SABA) as reliever medication. Patients only treated with a SAMA and/or SABA as well as patients on inhaled corticosteroids continued as before. Patients were instructed to not use any SAMA or SABA before visits 2 and 3. Thus, a morning visit meant no morning administration, and for an afternoon visit, only an early morning inhalation was allowed.

*Exercise testing*: The duration of exercise was collected at all exercise tests. Workload was registered at baseline, after 3–4 min and at the end of the tests. Borg dyspnea scale score and Borg leg discomfort scale score^20^ were measured every second minute. ECG and blood pressure were recorded before and after exercise. Ergospirometry was used for continuous measurement of VO_2_, VCO_2_, minute ventilation (VE) and respiratory rate (RR). Data were collected at 5-s intervals and processed in the following way: for each value of VO_2_, VCO_2_, VE and RR, the median of the five values encompassing the value was utilized. After exercise, the patients entered a recovery phase in the same way as during the standard tests.^6^

*ICPET*: After a few min of sitting on the bicycle to stabilize oxygen kinetics measurement equipment, the patients had an approximately 1-min warm-up period of loadless pedalling. After this, the patients started cycling (=time point 0) at a load of 40% of predicted *W_MAX_* for 4 min, followed by a linear increase in load, calculated to reach predicted *W_MAX_* after an additional 7 min. The linear increase in load was continued until the patients reached their measured *W_MAX_* at the point of exhaustion. The patients then entered a recovery phase with loadless pedalling (Figure 1).

*CET*: After a few min of sitting on the bicycle to stabilize oxygen kinetics measurement equipment, the patients had an approximately 1-min warm-up period of loadless pedalling. After this, the patients cycled for 3 min at a load of 40% of measured *W_MAX_* from visit 1, followed by an increase to 70% of measured *W_MAX_* from visit 1. After this, the load was linearly increased by 1.0% per min until the patients stopped due to exhaustion. The patients then entered a recovery phase with loadless pedalling (Figure 1).

### Study equipment

Patients performed flow-volume spirometry (MasterScreen, Erich Jaeger GmbH, Würzburg, Germany), body plethysmography (MasterScreen Body, Erich Jaeger GmbH), *DLCO* (MasterScreen PFT, Erich Jaeger GmbH) and ergospirometry (Oxycon Pro, Erich Jaeger GmbH). Established reference values by Crapo and colleagues^21^ were used.

Outcome measures

This study has separate outcome measures relating to the two different study objectives.

Part 1: Improvements of the prediction algorithm (studies A and B): The primary outcome of this part of the study was the development of a prediction algorithm based on pooled baseline demographic and lung function data from the *W_MAX_* tests performed in studies A and B with the objective of predicting *W_MAX_* with values of coefficient of determination (*R*^2^) and slope as close to 1.0 as possible.
Part 2: Bronchodilator effect on exercise performance (study C): The primary efficacy variable at the CET was endurance time measured after treatment with IND/GLY compared with placebo.

Secondary outcomes included work capacity (kWs), workload at end of exercise (W), Borg scale scores for dyspnea and leg discomfort, ergospirometric measurements and reasons for stopping exercise.

### Statistical analyses

We designed the studies to include 15–25 patients to obtain enough data in our development programme, but no formal power calculations were performed for either study.

In studies A and B,^17,18^ a model based on random forest regression was used for prediction of *W_MAX_*. In study C, this was changed to a model based on multiple regression. The reason for changing regression method was at least twofold: (1) the random forest methods require considerably more data/patients than standard regression methods ^22^ and we previously used data from 261 patients in a multicentre study.^6^ (2) Poor calibration may occur with the random forest method because the predictions by nature are biased away from the extreme values in the training dataset.^23^ This may explain why the predicted *W_MAX_* from the random forest algorithm underestimated the high-range performers and overestimated the low-range performers.

Multivariate regression with backward deletion was performed by stepwise removing the variable with the highest *p* value until only three variables remained. To construct the prediction algorithm, data from two-thirds of the patients (selected by random assignment) were used. Data from the remaining one-third of the patients were thereafter used for validation of the resulting prediction algorithm. SPSS v. 25 for Windows was used for multiple regression.

In study C, demographics and patient data were expressed as mean ± standard deviation unless otherwise stated. The normality of the continuous variables was checked by the Shapiro–Wilk test. The comparisons between predicted *W_MAX_* and measured *W_MAX_* were performed using univariate regression, Bland–Altman graphs and descriptive statistics. Microsoft Excel for Office 365 was used for univariate regression and Bland–Altman graphs. Endurance time was analysed using an analysis of variance (ANOVA) model with patient, period and treatment as fixed factors. A multiplicative model was used to check the stability of the results,^8^ complemented with an additive analysis to facilitate interpretation of the clinical importance of the results. Treatment differences were estimated from the model and 95% confidence intervals (CI) calculated. SPSS v. 25 for Windows was used for the Shapiro–Wilk test, and R v. 3.5.1 was used for ANOVA.

## Results

### Part 1

#### Improvement of the prediction algorithm for *W_MAX_*

*Patient data*. Table 1 presents baseline demographic and lung function data for the pooled dataset from study A + study B (*n* = 38). Patients in study B (*n* = 3), who also participated in study A, were removed from the pooled dataset. A flowchart of patients included in the pooled dataset is presented in Figure S1.

**Table 1.** Patient baseline characteristics per study for the pooled dataset and for study C.

Characteristic	Pooled dataset Study A + *B* (*n* = 38)	Study C (*n* = 14)
Age, years (range)	70 ± 6.1 (49–79)	61 ± 6.2 (47–67)
Male, *n* (%)	23 (61)	3 (21)
Height, cm	172 ± 9	170 ± 9
Weight, kg	78 ± 14	78 ± 12
BMI, kg/m^2^	26 ± 4	27 ± 3
Time since diagnosis, 1–5 years *versus* >5 years	8 *versus* 30	5 *versus* 9
Pack years, median (range)	33 (7–77)	28 (0–50)
*FVC*, L^a^	3.50 ± 0.93	3.33 ± 0.85
*FEV*_1_, L^a^	1.85 ± 0.48	1.95 ± 0.55
*FEV*_1_/*FVC*^a^	0.54 ± 0.09	0.59 ± 0.11
*FEV*_1_, % predicted^a^	65 ± 9.8	63 ± 14
*FVC*, % predicted^a^	92 ± 17	88 ± 12
*DLCO*, % predicted^a^	73 ± 19	77 ± 18
*IC*, % predicted^a^	80 ± 17	101 ± 34
*FRC*, % predicted^a^	119 ± 25	120 ± 26
Total CCQ score	0.99 ± 0.77	1.69 ± 0.96
CCQ symptom score	1.40 ± 1.03	2.11 ± 1.30
CCQ mental score	0.62 ± 0.77	1.75 ± 1.14
CCQ function score	0.84 ± 0.86	1.30 ± 0.90

BMI, body-mass index; CCQ, Clinical COPD Questionnaire; DLCO, diffusing capacity for carbon monoxide; FEV_1_, forced expiratory volume in 1 s; FRC, functional residual capacity; FVC, forced vital capacity; IC, inspiratory capacity.

Values are mean ± standard deviation unless otherwise specified.

a Postbronchodilator.

*New algorithm for the prediction of W_MAX_*. As described above in the ‘Statistical analyses’ section, multiple regression analyses were used for the development of a new prediction algorithm. Two other reasons to abandon the random forest prediction algorithm^17^ based on the multicentre study were (1) the regression slope for the algorithm constructed from multicentre data^6^ was 0.43 (intercept > 0) when applied to the pooled dataset (Table 2), that is, predicted *W_MAX_* values in the high and low range were underestimated and overestimated, respectively, and (2) one of the prime predictors, *DLCO*, showed univariate variability of *R*^2^ from 0.01 to 0.83^17^ between centres in the multicentre study,^6^ while our pooled data showed an *R*^2^ = 0.71 in univariate analyses, when baseline *DLCO* was plotted *versus* measured *W_MAX_* (Table 2).

**Table 2.** Development of the new prediction algorithm using multiple regression.

Model	*n*	Regression formula for predicted *W_MAX_*	*R* ^2^	Slope
Pooled dataset				
RF algorithm	38	See Eriksson and colleagues^17^	0.72	0.43
UR – *DLCO*	38	*DLCO*/0.042 − 1.48	0.71	1.00^a^
UR – *FEV*_1_	38	*FEV*_1_/0.0099 − 0.77	0.64	1.00^a^
UR – *Height*	38	*H*/0.118 + 159	0.26	1.00^a^
UR – *Age*	38	−*A*/0.045 + 75	0.08	1.00^a^
MR – (3 var) (2/3)	25	11.7*DLCO + 48.7*FEV_1_ − 0.827*H + 90.3	0.86	0.95
MR – (3 var) (1/3)	13	11.7*DLCO + 48.7*FEV_1_ − 0.827*H + 90.3	0.92	0.79
MR – (3 var) + *Age* + *Sex*	38	11.2*DLCO + 43.7*FEV_1_ − 1.00*H − 0.45*A + 13.2 (for males) + 157	0.89	0.89
Study C				
MR – (3 var) + *Age* + *Sex*	14	11.2*DLCO + 43.7*FEV_1_ − 1.00*H − 0.45*A + 13.2 (for males) + 157	0.91	0.82

A, age; DLCO, diffusing capacity for carbon monoxide; FEV_1_, forced expiratory volume in 1 s; H, height; MR, multivariate regression; RF, random forest; UR, univariate regression; 3 var (three variables) = *DLCO*, *FEV*_1_ and *height*.

Values of *R*^2^ and slope for plots of predicted *versus* measured *W_MAX_* have been presented based on pooled data from studies A and B, or data from study C (where noted). Results from univariate regression for the most important variables have also been included. Units: A: years; DLCO: ml/min/kPa; FEV_1_: L; H: cm.

a Slope from univariate regression is per definition = 1.00.

Therefore, we developed a new prediction algorithm based on the pooled dataset. In the validation, the patients were randomly divided into two-thirds (*n* = 25) and one-third (*n* = 13) of the patients. The larger and prime dataset was subjected to multiple regression with backward deletion, until only three variables remained. The resulting three-variable algorithm which included *DLCO*, *FEV*_1_ and *height* gave a value of *R*^2^ = 0.86 and slope = 0.95. Similar results were demonstrated for the smaller validation dataset: *R*^2^ = 0.92 and slope = 0.79 (Table 2).

After adding the variables *sex* and *age*, the following, final, new prediction algorithm was established

(1) $$\begin{array}{c} Predicted\;W_{MAX}=\;11.2*DLCO\;+\;43.7*FEV_{1}-\\ \;\;1.00*Height-0.45*Age\;+\;\\ \;\;13.2\;\left(for\;\;males\right)\;+\;157 \end{array}$$

As expected, higher values of *DLCO*, *FEV*_1_ and *sex* = male, and lower values for *age*, gave higher values of predicted *W_MAX_*. Multiple regression indicated a negative correlation between *height* and *W_MAX_*, but as expected, the univariate regression model showed a positive correlation. The negative correction factor for *height* in the multiple regression formula resulted partly from compensating for the strongly positive correction factor for *FEV*_1_ (because *height* is positively correlated with *FEV*_1_) and is a consequence of the fact that following multiple regression coefficients are estimated to give the best overall performance.

**Figure 2.**
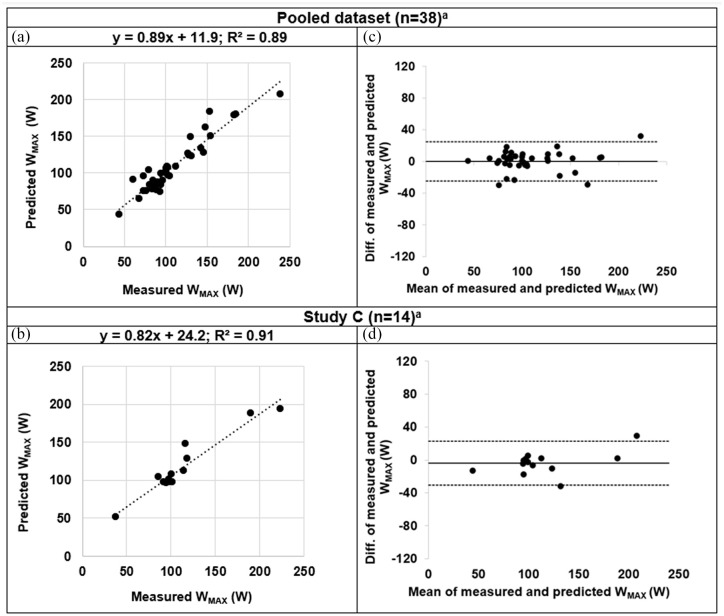
Correlation plots (a and b) and Bland–Altman graphs (c and d) of predicted *W_MAX_ versus* measured *W_MAX_*: predicted value from the new prediction algorithm was used in the pooled dataset (in a and c) and in study C (b and d). (a and b) Dotted line is line of identity. (c and d) Solid line shows the mean difference. Dotted lines show the limits of agreement, defined as the mean difference ± 1.96 SD of differences. DLCO, diffusing capacity for carbon monoxide; FEV_1_, forced expiratory volume in 1 s; SD, standard deviation. ^a^Included variables: *DLCO*, *FEV*_1_, *sex*, *age* and *height*.

In the pooled dataset, this new prediction algorithm resulted in a correlation between predicted *W_MAX_* and measured *W_MAX_* with an *R*^2^ = 0.89 and slope = 0.89 (Figure 2(a)). A Bland–Altman plot (Figure 2(c)) showed the agreement between predicted and measured *W_MAX_* giving a mean difference of 0.0 (SD: 12.8 and limits of agreement from −25.0 to 25.0).

The new prediction algorithm hereby showed better agreement between the predicted and measured *W_MAX_* than the initial random forest algorithm (which showed *R*^2^ = 0.43 and slope = 0.43, Figure S2A), when plotted in the same pooled dataset. The accompanying Bland–Altman plot (Figure S2B) showed a mean difference of 12.44 (SD: 17.9 and limits of agreement from −22.7 to 47.6).

### Part 2

#### Bronchodilator effect on exercise performance (study C)

*Patients in study C*. A total of 23 patients were enrolled between February and August 2019. Among these, 14 patients were included after fulfilling all inclusion criteria. Seven patients were excluded due to *FEV*_1_ ⩾ 80 %pred and two patients due to significant cardiovascular comorbidity. Included patients had a mean age of 61 years (range: 47–67 years), a mean *FEV*_1_ of 1.95 L (63 %pred) and mean *FEV*_1_/*FVC* ratio of 0.59. Demographics and further baseline characteristics are provided in Table 1. All continuous demographic and lung function baseline variables were normally distributed. No patients in study C participated in studies A or B.

*ICPET*. The 14 included patients performed the ICPET at visit 1. A plot of predicted *W_MAX_ versus* measured *W_MAX_* from study C is shown in Figure 2(b). The values for *R*^2^ = 0.91 and slope = 0.82 were similar to those obtained for the pooled dataset (Figure 2(a)). A Bland–Altman plot (Figure 2(d)) showed the agreement between predicted and measured *W_MAX_*, giving a mean difference of −3.6 (SD: 13.6 and limits of agreement from −30.3 to 23.1).

**Figure 3.**
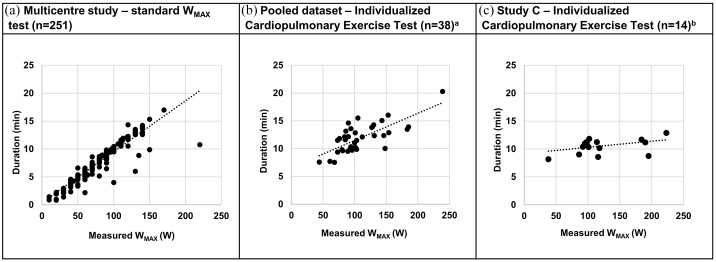
Plot of duration (min) *versus* measured *W_MAX_* for the standard *W_MAX_* test from the multicentre study (*n* = 261)^6^ (a), the ICPET from the pooled dataset (*n* = 38) (b) and the ICPET from study C (*n* = 14) (c). ^a^Initial *W_MAX_* from random forest algorithm; ^b^Initial *W^MAX^* from multiple regression algorithm. ICPET, individualized cardiopulmonary exercise test.

Plots of the exercise time *versus* measured *W_MAX_* of the standard *W_MAX_* test in the multicentre study,^6^ the ICPET in the pooled dataset and the ICPET in study C are shown in Figure 3. In the standard *W_MAX_* test from the multicentre study (Figure 3(a)), the exercise time was proportional to the work capacity of the patient, and a range of the exercise time from 1–17 min was observed. In the pooled dataset, using an initial *W_MAX_* calculated from the random forest algorithm, a flatter relationship between exercise time and work capacity was observed (Figure 3(b)). In study C (Figure 3(c)), using an initial *W_MAX_* calculated from the new prediction algorithm, the plot became almost horizontal and the time interval for the test procedure was more narrow (8–13 min) than in the standard *W_MAX_* test.

The results from the ICPET were similar in study C compared with the pooled dataset regarding *W_MAX_* reached, Borg scale scores and reasons for stopping exercise (Table 3). However, the range of the duration of the ICPET was more narrow in study C. Figure S3A gives graphical presentations of workload *versus* time for the individual patients during the ICPET.

**Table 3.** Results of the individualized cardiopulmonary exercise tests from the pooled dataset and from study C.

	Pooled dataset Study A + *B* (*n* = 38)	Study C (*n* = 14
*W_MAX_*, W	109 ± 39	112 ± 45
Range of *W_MAX_*, W	44–239	38–223
Time of exercise, min	11.9 ± 2.6	10.6 ± 1.3
Range of time of exercise, min	7.5–20.3	8.2–12.9
Borg dyspnea at end, score	7.2 ± 2.0	7.6 ± 2.1
Borg leg discomfort at end, score	16.6 ± 2.2	16.1 ± 3.5
Reason for stopping exercise, *n* (%)^a^		
Dyspnea	15 (39%)	7 (50%)
Dyspnea + Leg discomfort	14 (37%)	3 (21%)
Leg discomfort	8 (21%)	4 (29%)
Other reason	1^b^ (3%)	0

Values are mean ± standard deviation unless otherwise specified.

a Reported as dyspnea, leg discomfort, both of these or other reason.

b Stopping reason: bad bike seat.

### CET

**Figure 4.**
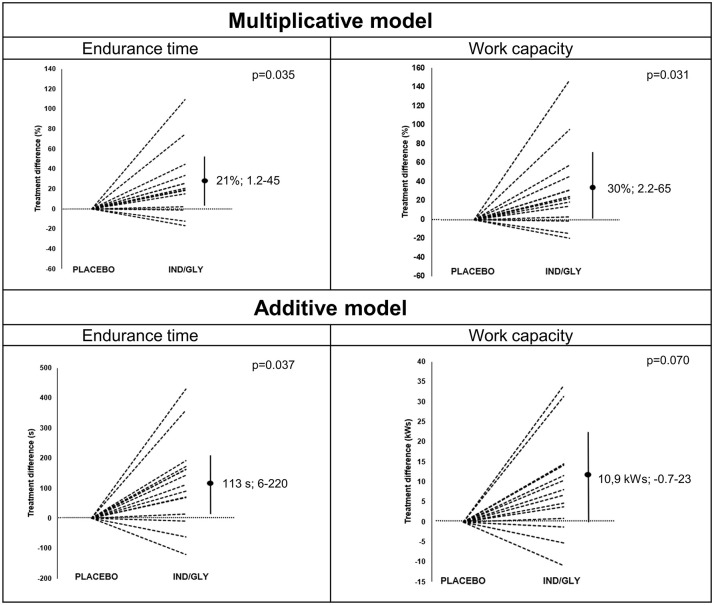
Graphical presentation of the results from the endurance tests (endurance time and work capacity) analysed by multiplicative or additive models, respectively. Treatment difference between IND/GLY and placebo per patient is shown with placebo normalized to zero. Dashed lines represent different individuals and vertical line represents 95% confidence interval with the dot showing the mean difference. IND/GLY, indacaterol/glycopyrronium.

The values for endurance time were normally distributed at both treatments, while the results for work capacity were not so at either treatment. Figure 4 gives a graphical presentation of the results from the CET performed at visits 2 and 3. Using a multiplicative model, statistical significance was observed for IND/GLY *versus* placebo for endurance time (difference 21%; 95% CI: 1.2–45%; *p* = 0.035). When using an additive model, again a statistically significant increase in endurance time was observed, with a mean difference of 113 s; 95% CI: 6–220 (*p* = 0.037).

Table 4 presents the results of the CET after treatment with IND/GLY and placebo, respectively, regarding endurance time, work capacity, ergospirometry, Borg scale scores and reasons for stopping exercise. Using the multiplicative model, a significant difference between treatments was observed for work capacity (difference 30%; 95% CI: 2.2-65%; p=0.031), but not for the peak VO2, VCO2, VE or RR levels nor the end Borg scale scores (Table S1). No important differences were observed for reasons for stopping exercise.

**Table 4.** Summarized results from the customized endurance tests in study C.

	IND/GLY (*n* = 14)	Placebo (*n* = 14)	*p* value^a^
*W_MAX_*, endurance time and work capacity			
*W_MAX_* reached, W	89 ± 38	87 ± 37	n.t.
Endurance time, min	11.1 ± 3.2	9.2 ± 2.8	0.035
Endurance time, s	665 ± 191	552 ± 166	0.035
Range time of exercise, min	7.1–16.9	5.6–14.1	n.t.
Work capacity, kWs [median (range)]	41 (21–158)	33 (11–128)	0.031
Ergospirometry			
Peak VO_2_, L/min	1.49 ± 0.52	1.38 ± 0.40	0.52
Peak VCO_2_, Ll/min	1.49 ± 0.56	1.36 ± 0.39	0.33
Peak VE, L/min	53 ± 18	47 ± 14	0.15
Peak RR, 1/min	34 ± 5	34 ± 6	1.00
Borg dyspnea and Borg leg discomfort scores			
Borg dyspnea at end, score	8.8 ± 1.8	7.6 ± 2.6	0.24
Borg leg discomfort at end, score	17.5 ± 2.8	16.6 ± 4.4	0.35
Reasons for stopping exercise			
Dyspnea, *n* (%)	6 (43%)	8 (57%)	n.t.
Dyspnea and leg discomfort, *n* (%)	3 (21%)	3 (21%)	n.t.
Leg discomfort, *n* (%)	5 (36%)	2 (14%)	n.t.
Other reason, *n* (%)	0	1 (7%)^b^	n.t.

IND/GLY, indacaterol/glycopyrronium; n.t., not tested; RR, respiratory rate; VE, minute ventilation.

Values are mean ± standard deviation unless otherwise specified.

a Multiplicative model.

b Stopping reason: dizziness.

Table S2 compares the results with those from study B. Figure S3B gives graphical presentations of workload *versus* endurance time for the individual patients during the CET for the IND/GLY treatment arm.

### Subanalyses based on baseline functional residual capacity (FRC) and *FEV*_1_

Explorative subanalyses, which were not prespecified, were performed to compare results from the CET for groups of patients with a baseline functional residual capacity (*FRC*) below and above 120 %pred and a baseline *FEV*_1_ below and above 70 %pred, respectively.

The baseline mean *FRC* %pred was 120% in study C. The difference in mean endurance time at the CET between IND/GLY and placebo for patients with an *FRC* ⩾ 120 %pred (*n* = 8) and <120 %pred (*n* = 6) was 144 and 78 s, respectively (see Figure S4A for plots of individual treatment differences *versus* baseline *FRC* %pred).

The baseline mean *FEV*_1_ %pred was 63 in study C. The difference in mean endurance time between IND/GLY and placebo was 177 s for patients with *FEV*_1_ ⩽ 70 %pred (*n* = 9, mean *FEV*_1_ %pred = 51), compared with −1 s for patients with *FEV*_1_ > 70% (*n* = 5). Figure S4B shows plots of treatment differences *versus* baseline *FEV*_1_ %pred.

## Discussion

Three studies have been performed in our development programme to improve the standard bicycle exercise test. We have compared our ICPET with the standard stepwise CPET in one study and our CET with the CWRET in two studies. These comparisons showed similar results, but the objective of lower standard deviation for time to exhaustion was met for both ICPET and CET, thus showing construct validity. In addition, both the ICPET and CET showed generalizability, as tested in three studies with different patients and with the same inclusion criteria, that is, external validity. Furthermore, in this study, our CET showed a significant responsiveness of endurance time for IND/GLY and hence also providing evidence for internal/criterion validity. Taken together, these results support the usefulness of the combined ICPET and CET to assess bronchodilator treatment effects on exercise performance.

The new prediction algorithm used pooled data (*n* = 38) from our previous single-centre studies^17,18^ and was based on a multiple regression with backward deletion model instead of the previously used random forest method. *FEV*_1_ and *DLCO* were found to be the best predictors which together with *sex*, *age* and *height* were used in the equation. A prediction algorithm based on results from a multicentre study may be more generalizable than if constructed on data from a single-centre study. However, in a multicentre study by Maltais and colleagues,^6^ the primary objective was to study the endurance time, and these primary and secondary objectives were probably more closely monitored than the predictors used in our algorithm. For example, a large intercentre variability was observed for *DLCO* (only recorded as baseline parameter) as a function of measured *W_MAX_*.^6^ In our single-centre studies, the procedures for ICPET and CET, as well as the baseline recordings, were closely monitored with the intention to find new predictors and an improved prediction algorithm for *W_MAX_*. Test results collected from only one qualified study centre can thus be expected to be exposed to a lower risk of quality problems and abnormal spread as compared with results from multicentre clinical trials. An important learning is, however, that when using our prediction algorithm in future settings, high quality of the defined predictors, for example, *FEV*_1_ and *DLCO*, must be standardized to the same quality as the procedure of the exercise tests.

The new prediction algorithm based on the pooled dataset showed a high coefficient of determination and a slope close to 1 for predicted *versus* measured *W_MAX_*. Using this prediction algorithm in this study resulted in an *R*^2^ of 0.91 and a slope of 0.82 for predicted *versus* measured *W_MAX_*. This provides strong support for the validity of this approach for the prediction of *W_MAX_* and an accurate determination of the patients’ measured *W_MAX_* when using the ICPET.

A major advantage over a standard *W_MAX_* test is that the ICPET is individualized. In the ICPET, COPD patients with a low predicted *W_MAX_* are given a lower increase in workload/min than those with a high predicted *W_MAX_*. The difference in *W_MAX_*/min between patients with different predicted *W_MAX_* values is because exercise time to *W_MAX_* is precalculated to a fix length in the ICPET. This is different from a standard maximum test, where all patients face identical incremental increases. Debigare and colleagues^24^ demonstrated in the stepwise standard exercise test that a low increase of workload/min resulted in lower *W_MAX_*, whereas a high increase of workload/min resulted in a higher *W_MAX_*. This phenomenon may affect our ICPET because different workload/min were applied based on the individual fixed escalation protocol. This effect may be relevant for the first prediction algorithm, as being based on standard CPET data and applied in the new ICPET. However, the new prediction algorithm was constructed from the same patient’s baseline data and *W_MAX_* value derived from ICPET, that is, the effect by Debigare and colleagues^24^ is included in the prediction algorithm and thereby not relevant for our exercise test. The end result of an improved ICPET including the new prediction algorithm may lead to a more accurate measured *W_MAX_* to be used in the CET. The more narrow interval to reach *W_MAX_* may also facilitate an improved quality and focus for the patients and the study personnel.

In the subsequent CET, the endurance performance derived from the ICPET in study C may benefit with a more accurate *W_MAX_* value (see above). The 70% start in the endurance test in studies B and C, compared with 75% in study A, may facilitate for low performers to cycle longer. Similarly, the 1% per minute of escalation will force high performers to face an earlier exhaustion than with the standard test. Decreasing the coefficient of variation for endurance time has been shown to diminish the required sample size of the study.^11,25^

The CET showed a statistically significant prolongation of endurance time for IND/GLY *versus* placebo (113 s) in a crossover study in patients with moderate to severe COPD. The improvement in endurance time is above the minimally clinically important difference for submaximal exercise endurance time on a cycle ergometer of 46–105 s as suggested.^15^ However, the clinical importance of the improvement of 113 s with IND/GLY shown in this study should be interpreted with some caution. Statistically significant improvement was seen also for work capacity (30%), which could provide some additional support for a benefit of IND/GLY on exercise performance compared with placebo. Regarding ergospirometry and Borg scale scores, numerically higher values were observed for IND/GLY *versus* placebo for all parameters except RR, but no significant treatment differences were found. No differences were observed between IND/GLY and placebo for reasons for stopping exercise.

How do our results regarding the primary outcome endurance time relate to other published exercise studies between fixed combination LABA/LAMA and placebo using a standard CWRET? We found one single-dose study with tiotropium/olodaterol *versus* placebo reporting a significant increase in endurance time of 88 s in a highly selected patient population.^26^ Two other long-term studies with LAMA/LABA *versus* placebo reported increases in endurance time of 24 s^27^ after the initial dose and 40 s^28^ after the second dose on day 2, respectively. We also found five studies comparing endurance time between different LABA/LAMA medications with placebo over 3–12 weeks.^27–31^ The differences in endurance time between LABA/LAMA treatment and placebo at the end of treatment ranged from 55 to 85 s. Overall, the improvement in endurance time after single-dose treatment with IND/GLY in our CET seems to be numerically larger than in similar studies comparing LABA/LAMA treatment with placebo using the standard CWRET.^32^

An important factor when comparing results between studies are differences in inclusion criteria. Additional criteria such as signs of lung hyperinflation, often defined as an increase of *FRC* ⩾ 120 %pred,^5,6,26,33^ have been used in studies of treatment effects of bronchodilators on exercise performance in patients with COPD. An *FRC* ⩾ 120 %pred was also used for selection of the study population in three of the aforementioned studies.^26,28,31^ In addition, a study comparing IND/GLY *versus* placebo demonstrated an increase in endurance time of 60 s in all patients compared with 86 s in the subgroup of patients with *FRC* ⩾ 120 %pred.^27^ Similarly, this study showed a more pronounced effect of IND/GLY on endurance time in patients with *FRC* ⩾ 120 %pred compared with <120 %pred, but the number of patient in each group was limited. This benefit of selecting more responsive patients has been described by Di Marco and colleagues^34^ in a systematic review of long-acting bronchodilators.

Another important selection criterion is *FEV*_1_ %pred. In the studies above, there is a range for the lower and higher *FEV*_1_ level of 30–40 and 70–80 %pred, respectively, resulting in a wide range of the mean *FEV*_1_ of 47–63 %pred. As our mean *FEV*_1_ %pred was the highest within all studies, we performed a subanalysis applying a limit of *FEV*_1_ ⩽ 70%pred. A more pronounced effect of IND/GLY on endurance time was shown in patients with *FEV*_1_ ⩽ 70 %pred compared with >70 %pred, but the number of patients in each group was limited. The finding of a longer endurance time at lower *FEV*_1_ %pred is supported by a study by O’Donnell and colleagues^35^ where the difference between indacaterol and placebo was 229 s for *FEV*_1_ < 50 %pred (*p* < 0.05) and 85 s (nonsignificant) for *FEV*_1_ ⩾ 50 %pred. A cut-point at 70 %pred is in line with breakpoints for different lung function parameters as a function of *FEV*_1_ %pred, where, on a group level, measures of hyperinflation [e.g. residual volume (RV), FRC, and total lung capacity (TLC)] increase and *DLCO* decreases below the breakpoint, while only limited changes occur above.^36^ In addition, the bronchodilator response of several advanced lung function parameters is most pronounced in COPD patients with an *FEV*_1_ %pred of ⩽65 compared with >65, specifically the bronchodilator response of volume parameters.^37^

A limitation to our study is that the results may only be applicable to COPD patients with *FEV*_1_ %pred between 40% and 80%, and not be generalizable to other COPD patient populations, for example, patients with cardiovascular symptoms. For example, coexisting pulmonary hypertension may have an impact on *W_MAX_* and endurance time but was not investigated in this study. However, the crossover study design makes it unlikely that presence of pulmonary hypertension would have had any significant impact on the results.

The size of this study was limited to 14 patients, why the results should be interpreted with some caution, and particularly those from subanalyses regarding *FRC* and *FEV*_1_. Such small subanalyses should only be regarded as hypothesis generating, although showing convincing results supported by published data. During the process of optimizing our methodology, the study protocols for both the ICPET and CET have been subjected to small modifications, for example, the initial workload in the ICPET used either 30% or 40% during 3 or 4 min together with 8- or 7-min escalation period, giving a total of 11 min.^17,18^

Conclusions drawn from comparisons between studies should always be done with great caution. This is illustrated in the work by Puente-Maestu and colleagues,^14^ where a wide range of differences are presented for different bronchodilators.

The next step would be to repeat the study in more patients with more centres and over longer treatment times. In such a study, the question about selection criteria will arise. Should it be with or without an *FRC* ⩾ 120 %pred or is the answer a lowering of *FEV*_1_ from ⩽80 to ⩽70 %pred?^36,37^ Our small explorative subanalyses indicate the latter. In future studies, it may also be possible to replace the ICPET with a baseline CET (using an initial load based on the predicted *W_MAX_*) to reduce a potential increase in cardiac risks associated with maximum tests^9,10^ and decrease the risk for training effects during the blinded crossover periods.

The objective of this project was to improve the standard CWRET by reducing the variation in endurance time among patients, leading to fewer patients needed to detect differences between treatments. We have in three sequential studies achieved a better prediction algorithm, an individualized linear maximal test with a low range of the variability in the exercise time and an endurance test which by design avoid short and long durations. In addition, the CET has been reproduced and showed a significant and clinically relevant prolongation of endurance time of IND/GLY, supported by significant increases of total work capacity. Together, these results provide support that we have achieved the overall goal of our development programme of an improved exercise testing protocol (including an ICPET and a CET). Our results should be confirmed in a prospective study with a larger number of patients.

## Supplemental Material

sj-docx-1-tar-10.1177_17534666211037454 – Supplemental material for A new protocol for exercise testing in COPD; improved prediction algorithm for WMAX and validation of the endurance test in a placebo-controlled double bronchodilator study Click here for additional data file. Supplemental material, sj-docx-1-tar-10.1177_17534666211037454 for A new protocol for exercise testing in COPD; improved prediction algorithm for WMAX and validation of the endurance test in a placebo-controlled double bronchodilator study by Ellen Tufvesson, Finn Radner, Anton Simonsen, Georgia Papapostolou, Linnea Jarenbäck, Saga Jönsson, Ulf Nihlen, Alf Tunsäter, Jaro Ankerst, Stefan Peterson, Leif Bjermer and Göran Eriksson in Therapeutic Advances in Respiratory Disease
