# Supplementary material for: A new protocol for exercise testing in COPD; improved prediction algorithm for WMAX and validation of the endurance test in a placebo-controlled double bronchodilator study
Source: Ther Adv Respir Dis. 2021 Sep 30;15:17534666211037454. doi: 10.1177/17534666211037454 (PMC8488527; doi:10.1177/17534666211037454)
Supplement: sj-docx-1-tar-10.1177_17534666211037454 – Supplemental material for A new protocol for exercise testing in COPD; improved prediction algorithm for WMAX and validation of the endurance test in a placebo-controlled double bronchodilator study [file sj-docx-1-tar-10.1177_17534666211037454.docx]

On-line supplement

**A new protocol for exercise testing in COPD; improved prediction algorithm for W_MAX_ and validation of the endurance test in a placebo-controlled double bronchodilator study.**

Ellen Tufvesson^1^, Finn Radner^1^, Anton Simonsen^1^, Georgia Papapostolou^1^, Linnea Jarenbäck^1^, Saga Jönsson^1^, Ulf Nihlen^1^, Alf Tunsäter^1^, Jaro Ankerst^1^, Stefan Peterson^2^, Leif Bjermer^1^ and Göran Eriksson^1^.

Figure S1. Flow chart of the preparation of the pooled dataset.


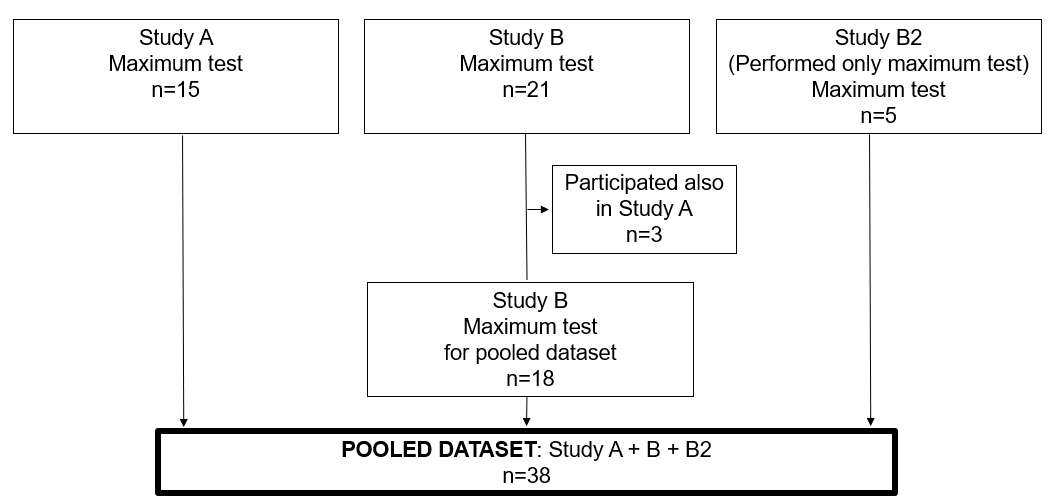


Figure S2. Correlation plot (A) and Bland-Altman graph (B) of predicted W_MAX_ *versus* measured W_MAX_ from the random forest algorithm for the pooled dataset (A and B).

| **Pooled dataset (n=38)^a^ Random forest algorithm** | |
| --- | --- |
| **A y = 0.43x + 56.9; R² = 0.43** | **B** |
|  |  |

**Notes**: ^a^ Included variables: Sex, Age, Height, DLCO, FEV_1_, FEF_50_, FVC, VA and FEF_25-75_; A: Dotted line is line of identity; B: Solid line shows the mean difference. Dotted lines show the limits of agreement, defined as the mean difference ± 1.96 SD of differences.

**Abbreviations:** DLCO, diffusing capacity for carbon monoxide; FEV_1_, forced expiratory volume in 1 second; FEF_50_, forced expiratory flow at 50%; forced vital capacity, FVC; VA, alveolar volume; mid-expiratory flow, FEF_25-75_; SD, standard deviation.

Figure S3. Individual patient graphs of work load *versus* duration during the Individualized Cardiopulmonary Exercise Test (A) and work load *versus* endurance time for Customized Endurance Test (IND/GLY treatment arm) in Study C (B).

| **A: Individualized Cardiopulmonary Exercise Test** | **B: Customized Endurance Test** **– IND/GLY treatment arm** |
| --- | --- |
|  |  |

**Abbreviations**: IND/GLY, indacaterol /glycopyrronium

Figure S4. Plots of treatment difference in endurance time *versus* baseline FRC %pred (A) and FEV_1_ %pred (B) in Study C. Vertical dotted lines represents baseline FRC %pred = 120% and FEV_1_ %pred = 70%, respectively.

| **A: Treatment difference vs baseline FRC %pred** | **B: Treatment difference vs baseline FEV_1_ %pred** |
| --- | --- |
| 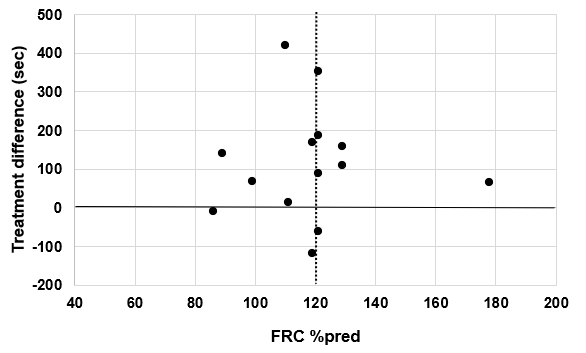 | 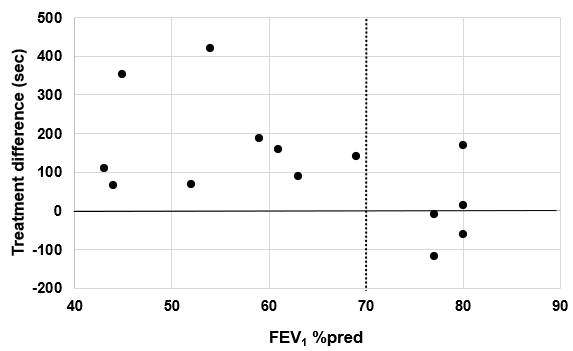 |

**Abbreviations**: FRC, functional residual capacity; FEV_1_, forced expiratory volume in 1 second

Table S1. Comparison between treatment with IND/GLY and treatment with placebo for Study C.

| **Variable** | **Treatment or treatment difference** | **Estimate/difference** | **95% confidence interval** | **p-value** |
| --- | --- | --- | --- | --- |
| **Multiplicative model** | | | | |
| Endurance time, min | IND/GLY | 10.7^a^ | 9.39-12.1 |  |
|  | PLA | 8.81^a^ | 7.77–9.99 |  |
|  | IND/GLY – PLA | 1.21^b^ | 1.01-1.47 | 0.035 |
|  |  |  |  |  |
| Work capacity, kWs | IND/GLY | 44.5^a^ | 37.6–52.6 |  |
|  | PLA | 34.3^a^ | 29.0–40.6 |  |
|  | IND/GLY - PLA | 1.30^b^ | 1.02-1.65 | 0.031 |
|  |  |  |  |  |
| Peak VO_2_, L/min | IND/GLY - PLA | 1.06^b^ | 0.95-1.17 | 0.52 |
| Peak VCO_2_,Ll/min | IND/GLY - PLA | 1.07^b^ | 0.96-1.20 | 0.33 |
| Peak VE, L/min | IND/GLY - PLA | 1.13^b^ | 0.97-1.32 | 0.15 |
| Peak RR, min^-1^ | IND/GLY - PLA | 1.00^b^ | 0.90-1.10 | 1.00 |
| Borg dyspnea at end, score | IND/GLY - PLA | 1.26^b^ | 0.90–1.77 | 0.24 |
| Borg leg discomfort at end, score | IND/GLY - PLA | 1.09^b^ | 0.95-1.24 | 0.35 |
|  |  |  |  |  |
| **Additive model** | | | | |
| Endurance time, s | IND/GLY | 665^c^ | 589–740 |  |
|  | PLA | 552^c^ | 476–627 |  |
|  | IND/GLY – PLA | 113^d^ | 6–220 | 0.037 |
|  |  |  |  |  |
| Work capacity, kWs | IND/GLY | 53.2^c^ | 44.9–61.4 |  |
|  | PLA | 42.2^c^ | 33.9–50.5 |  |
|  | IND/GLY - PLA | 10.9^d^ | -0.75-22.6 | 0.070 |

**Notes:** ^a^ Exponentiated adjusted mean; ^b^ Ratio of the estimates for IND/GLY and PLA; ^c^ Adjusted mean; ^d^ Treatment difference between IND/GLY and PLA (in s or kWs).

**Abbreviations**: IND/GLY, indacaterol /glycopyrronium; PLA, placebo; VE, minute ventilation; RR, Respiratory rate.

Table S2. Results from the Customized Endurance Tests in Study B and Study C.

| **Variable** | **Study B**  **n=18** | **Study C; IND/GLY n=14** | **Study C; PLA n=14** |
| --- | --- | --- | --- |
| **Workload** | | | |
| Workload at start, W^a^ | 30 ± 9 | 45±18 | 45±18 |
| Workload at 3 min, W | 70 ± 23 | 79± 31 | 79± 31 |
| Workload at end, W | 78 ± 27 | 89± 38 | 87± 37 |
| SD of workload at end, % | 34% | 42% | 43% |
| Range of workload at end, W | 33–146 | 30–186 | 28–180 |
| **Time of exercise** | | | |
| Endurance time, min | 10,7 ± 3,0 | 11,1 ± 3,2 | 9,2 ± 2,8 |
| SD in % of endurance time | 28% | 29% | 30% |
| Median endurance time, min | 9,4 | 10,9 | 8,9 |
| Range of endurance time, min | 7,4–16,3 | 7,1–16,9 | 5,6–14,1 |
| **Work capacity** | | | |
| Mean work capacity, kWs | 42 | 51 | 42 |
| Median work capacity, kWs | 34 | 41 | 33 |
| Range of work capacity, kWs | 12–97 | 21–158 | 11–128 |

**Notes:** Values are mean ± standard deviation unless otherwise specified. ^a^ Workload at start = 30% (Study B) or 40% (Study C) of the measured WMAX from the preceding Individualized Cardiopulmonary Exercise Test.
